# Tissue-type plasminogen activator modulates macrophage M2 to M1 phenotypic change through annexin A2-mediated NF-κB pathway

**DOI:** 10.18632/oncotarget.21510

**Published:** 2017-10-04

**Authors:** Ling Lin, Kebin Hu

**Affiliations:** ^1^ Department of Cellular and Molecular Physiology, Penn State University College of Medicine, Hershey, Pennsylvania, USA; ^2^ Division of Nephrology, Department of Medicine, Penn State University College of Medicine, Hershey, Pennsylvania, USA

**Keywords:** serine protease, tissue-type plasminogen activator, M1 macrophages, M2 macrophages, polarity shift

## Abstract

Macrophage accumulation is one of the hallmarks of progressive kidney disease. In response to injury, macrophages undergo a phenotypic polarization to become two functionally distinct subsets: M1 and M2 macrophages. Macrophage polarization is a dynamic process, and recent work indicates that macrophages, in response to kidney injury, can shift their polarity. However, the underlying mechanisms remain largely unknown. Tissue-type plasminogen activator (tPA), a protease up-regulated in the chronically injured kidneys, has been shown to preferably promote M1 macrophage accumulation and renal inflammation. We hypothesized that tPA may be an endogenous factor that modulates macrophage M2 to M1 phenotypic change contributing to the accumulation of M1 macrophages in the injured kidneys. It was found that obstruction-induced renal M1 chemokine expression was alleviated in tPA knockout mice, and these knockout mice displayed increased M2 markers. *In vitro*, resting J774 macrophages were treated with IL-4 to induce M2 phenotype as indicated by de novo expression of arginase 1, Ym1, and IL-10, as well as suppression of iNOS, TNF-α, and IL-1β. Intriguingly, these IL-4-induced M2 macrophages, after tPA treatment, not only lost their M2 markers such as arginase 1, Ym1, and IL-10, but also displayed increased M1 chemokines including iNOS, TNF-α, and IL-1β. Possible endotoxin contamination was also excluded as heat-inactivated tPA lost its effect. Additionally, tPA-mediated macrophage M2 to M1 phenotypic change required its receptor annexin A2, and SN50, a specific NF-κB inhibitor, abolished tPA's effect. Thus, it's clear that tPA promotes macrophage M2 to M1 phenotypic change through annexin A2-mediated NF-κB pathway.

## INTRODUCTION

Regardless of the initial causes, macrophage accumulation is one of the histological hallmarks of most interstitial and glomerular kidney diseases [1, 2]. Sustained macrophage accumulation in the damaged kidneys eventually becomes pathological, resulting in irreversible fibrosis, tissue destruction, and progressive chronic kidney disease (CKD) [1]. As a key component of innate immunity, macrophages are actually heterogeneous cells. In response to pathogenic cues, macrophages are differentiated into two broad but distinct subsets that are categorized as either classically activated (M1) or alternatively activated (M2) [1]. During classical activation, M1 macrophages express a panoply of proinflammatory genes to promote inflammation and damage through a combination of transcription factors, including NF-κB, and mitogen-activated protein kinases (MAPKs) [3]. In contrast, M2 macrophages, differentiated from alternative activation, help to resolve inflammation and promote tissue remodeling. Emerging evidence indicates that macrophages can switch their phenotypes between M1 and M2 in the diseased kidneys [4, 5]. However, the underlying mechanisms remain largely unknown.

tPA, a member of the serine protease family, has been shown to act as a cytokine to promote the kidney fibrosis and inflammation by triggering profound receptor-mediated intracellular signaling events [6–15]. Our recent work demonstrated that myeloid-derived tPA activates NF-κB, induces the accumulation of M1 macrophages, and promotes renal inflammation [12, 14, 15]. It is reasonable to suspect that M2 to M1 phenotypic change contributes to M1 macrophage accumulation. Recent work supports the notion that tissue microenvironment determines macrophage phenotypic polarization [16, 17], suggesting that localized factors in the injured kidneys may modulate the phenotypic shift of resident macrophages. Thus, it's presumable that tPA, a protease up-regulated in the kidneys with chronic injury, may be the endogenous factor that regulates macrophage M2 to M1 phenotypic change that leads to the accumulation of M1 macrophages in the injured kidneys.

In the present study, we investigated the role of tPA in macrophage M2 to M1 phenotypic change and elucidated the underlying signaling mechanisms using both *in vitro* and *in vivo* approaches. Our data demonstrate that tPA promotes macrophage M2 to M1 phenotypic change through annexin A2-mediated NF-κB pathway.

## RESULTS

### tPA promoted M1 macrophage phenotype in the obstructed kidneys

**Figure 1:**
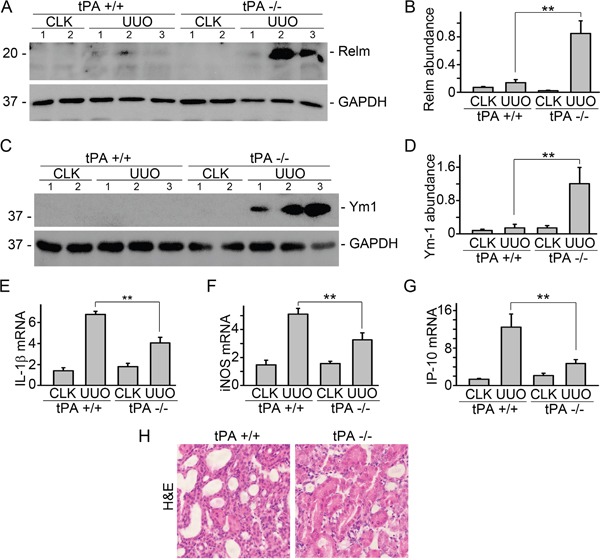
tPA promoted M1 macrophage phenotype in the obstructed kidneys UUO was performed in tPA WT and KO mice, followed by Western blot for Relm-α **(A)** and Ym1 **(C)**. Quantitative illustration of Relm-α **(B)** and Ym1 **(D)** abundance, n=5, ^**^P < 0.01, number indicates individual mouse, UUO 7d. **(E)** Quantitative IL-1β mRNA, n=3, ^**^P < 0.01, UUO 3d. **(F)** Quantitative iNOS mRNA, n=3, ^**^P < 0.01, UUO 3d. **(G)** Quantitative IP-10 mRNA, n=5, ^**^P < 0.01, UUO 14d. **(H)** H&E staining, UUO 7d. CLK: contralateral unobstructed kidney.

To examine the role of tPA in macrophage polarity *in vivo*, unilateral ureter obstruction (UUO), a classical model of CKD, was induced in tPA knockout (KO) and wildtype (WT) mice. Western blot results showed that M2 macrophage markers, such as Relm-α and Ym1, were dramatically induced in the obstructed kidneys from tPA KO mice than those from their WT counterparts (Figure 1A-1D). Induction of M1 chemokines, such as IL-1β (Figure 1E), iNOS (Figure 1F), and IP-10 (Figure 1G), in the WT mice was significantly reduced in tPA KO mice. In consistent with our previous work, WT mice displayed more severe kidney injury than tPA KO mice (Figure 1H) [6, 8, 10, 12, 14, 15, 18]. These results, in combination with our previous finding that WT mice had dramatically higher number of TNF-α^+^CD11b^+^ or CD11b^+^F4/80^lo^CD206^−^ M1 macrophages than their KO littermates [14], further confirmed that tPA preferably promotes M1 macrophages accumulation in the diseased kidneys after obstructive injury.

### tPA induced phenotypic change of IL-4-induced M2 macrophages to M1

**Figure 2:**
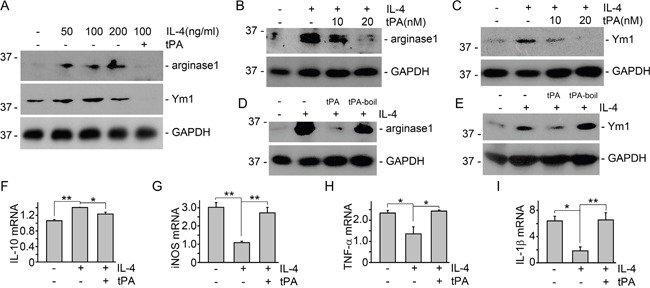
tPA induced macrophage M2 to M1 phenotypic change *in vitro* **(A)** J774 cells were treated with IL-4 at the concentrations as indicated in the presence of vehicle or 20 nM tPA for 24 h, followed by Western blot for arginase 1, Ym1, and GAPDH. **(B** and **C)** J774 macrophages were treated with 100 ng/ml IL-4 for 24 h with vehicle or tPA (10 and 20 nM), followed by Western blot for arginase 1 (B) and Ym1 (C). **(D** and **E)** IL-4-treated J774 macrophages were also incubated with tPA or heat-inactivated tPA (tPA-boil). Cell lysates were then probed for arginase 1 (D), Ym1 (E), and GAPDH. **(F-I)** mRNAs were extracted from J774 macrophage treated with 100 ng/ml IL-4 and vehicle or 20 nM tPA for 24 h, followed by quantitative PCR assay for IL-10 (F), iNOS (G), TNF-α (H), and IL-1β (I), n=3, ^*^P < 0.05, ^**^P < 0.01.

Macrophage polarization is a dynamic process, and recent observations indicate that macrophages can switch their phenotypes between M1 and M2 in the damaged kidneys at different disease stages [4, 5]. Thus, in addition to the balance between expansion through proliferation and clearance by apoptotic death [1, 14, 19], tPA-mediated M2 to M1 phenotypic change may also contribute to the accumulation of M1 macrophages in the damaged kidneys. To test our hypothesis, resting J774 macrophages were treated with IL-4, a classic M2 macrophage stimulator, to induce M2 phenotype. As shown in Figure 2, IL-4 induced M2 phenotype in J774 macrophages largely in dose-dependent manner, as demonstrated by induction of M2 markers of arginase 1, Ym1, and IL-10; and suppression of M1 chemokines including iNOS, TNF-α, and IL-β. However, tPA dose-dependently reduced IL-4-induced M2 makers of arginase 1 (Figure 2B) and Ym1 (Figure 2C). Moreover, tPA also reversed the M2 chemokine profile of IL-4-treated macrophages to M1 phenotype, as demonstrated by reduced IL-10 expression (Figure 2F) and up-regulation of iNOS (Figure 2G), TNF-α (Figure 2H), and IL-1β (Figure 2I). Possible contamination of endotoxin was also excluded because heat-inactivated tPA lost its effect (Figure 2D and 2E). Thus, it's clear that tPA promoted macrophage M2 to M1 phenotypic change.

### tPA-mediated macrophage M2 to M1 phenotypic change required annexin A2 but not LRP-1

**Figure 3:**
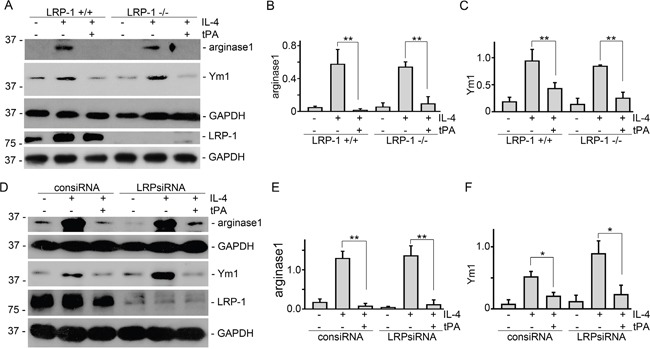
LRP-1 did not mediate tPA-induced macrophage M2 to M1 phenotypic change Primary bone marrow macrophages from LRP-1 WT or KO mice **(A-C)**, and J774 macrophages transfected by control or LRP-1 siRNAs **(D-F)** were treated with 100 ng/ml IL-4 with or without 20 nM tPA for 24 h, followed by Western blot (A and D) for arginase 1, Ym1, LRP-1, and GAPDH. Quantitative illustration of arginase 1 (B and E) and Ym1 (C and F) abundance, n=3, ^*^P < 0.05, ^**^P < 0.01.

**Figure 4:**
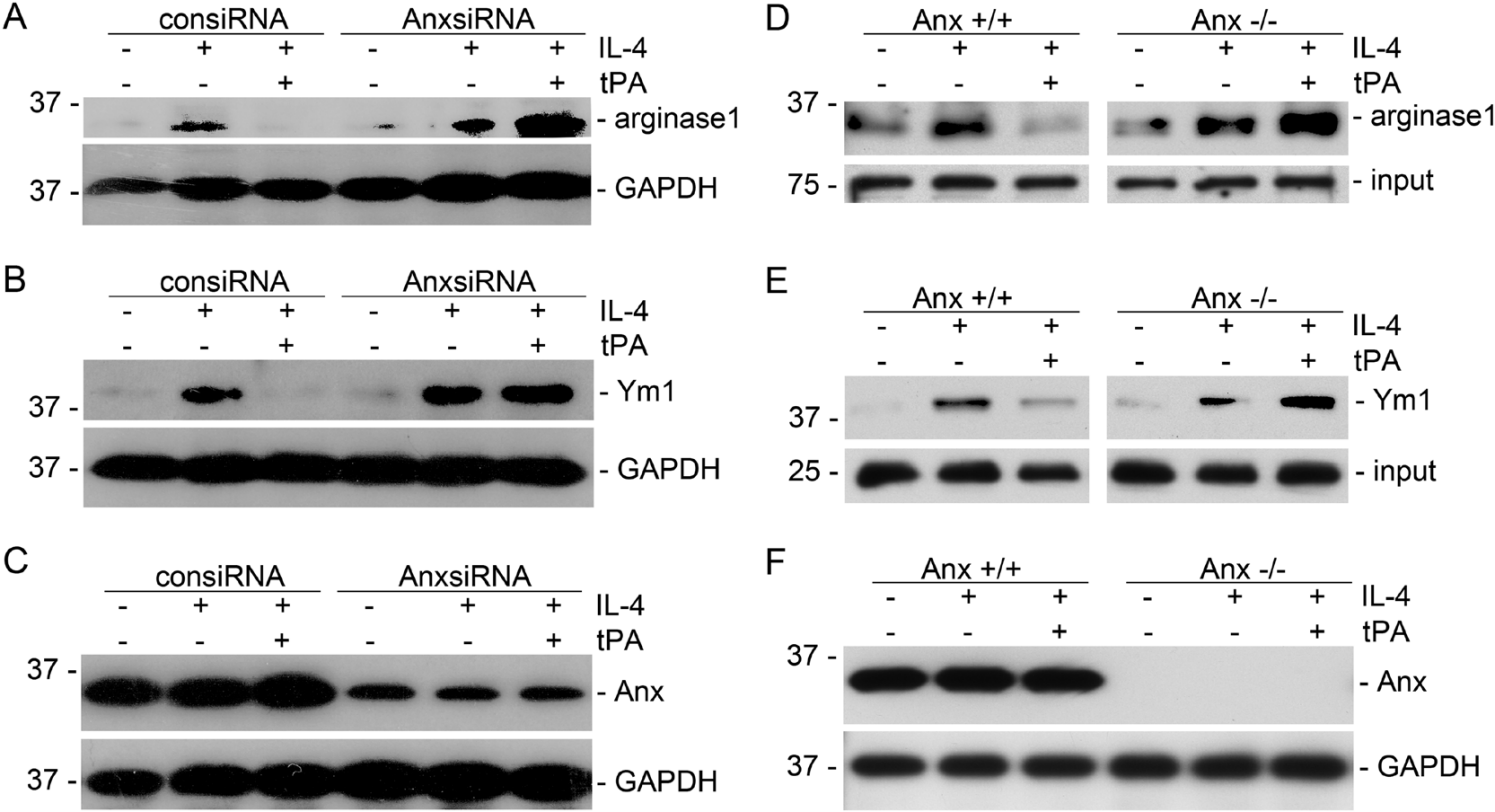
Annexin A2 mediated tPA-induced macrophage M2 to M1 phenotypic change J774 macrophages transfected by control or annexin A2 siRNAs **(A-C)**, and primary bone marrow macrophages from annexin A2 WT or KO mice **(D-F)** were treated with 100 ng/ml IL-4 with or without 20 nM tPA for 24 h, followed by Western blot for arginase 1 (A and D), Ym1 (B and E), annexin A2 (C and F), and GAPDH.

Our previous work showed that tPA modulated macrophage function and differentiation through either LRP-1 or annexin A2 [12, 14, 15]. We used both siRNA knockdown and knockout primary bone marrow macrophages to determine which receptor mediates the actions of tPA. It's found that tPA retained its ability to reduce M2 markers of arginase 1 and Ym1 in IL-4-treated primary bone marrow macrophages from LRP-1 KO mice (Figure 3A-3C) or in LRP-1 siRNA knockdown J774 macrophages (Figure 3D-3F). The KO or knockdown of LRP-1 was confirmed by Western blots (Figure 3A and 3D). Thus, LRP-1 did not mediate tPA-induced M2 to M1 phenotypic change. In contrast, tPA failed to induce M2 to M1 phenotypic change in either annexinA2 siRNA knockdown (Figure 4A-4C) or KO (Figure 4D-4F) macrophages. Therefore, annexin A2 was essential to tPA-induced M2 to M1 macrophage phenotypic change.

### NF-κB mediated tPA-induced macrophage M2 to M1 phenotypic change

**Figure 5:**
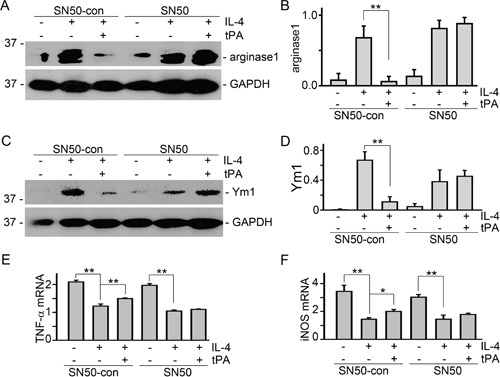
NF-κB was indispensable to tPA-induced macrophage M2 to M1 phenotypic change **(A** and **B)** J774 macrophages were pretreated with SN50 and its control peptide (50 μg/ml) for 30 min followed by incubation with 100 ng/ml IL-4 plus vehicle or 20 nM tPA for 24 h. Then, cell lysates were probed for arginase 1 (A), Ym1 **(C)**, and GAPDH. Quantitative illustration of arginase 1 (B) and Ym1 **(D)** abundance, n=3, ^**^P < 0.01. **(E** and **F)** mRNAs were extracted from J774 cells with above treatment for 24 h, followed by quantitative PCR assay for TNF-α (E) and iNOS (F), n=3, ^*^P < 0.05, ^**^P < 0.01.

Our recent work demonstrated that tPA activates NF-κB signaling in macrophages [14] and NF-κB mediates tPA-induced macrophage migration [15]. To determine the role of NF-κB in tPA-induced M2 to M1 phenotypic change, IL-4-treated M2 macrophages were treated with cell-permeable NF-κB-specific inhibitory peptide SN50 and its control peptide SN50-con [15]. It's found that tPA-induced M2 to M1 phenotypic change, as demonstrated by reduction of arginase 1 (Figure 5A and 5B) and Ym1 (Figure 5C and 5D), and induction of TNF-α (Figure 5E) and iNOS (Figure 5F), was abolished by SN50 (50 μg/ml). This result further confirmed that NF-κB is indispensable to tPA-induced macrophage M2 to M1 phenotypic change.

## DISCUSSION

One of the histological characteristics of both acute kidney injury and CKD is interstitial macrophage accumulation. Macrophages, in response to persistent injury, accumulate within tissue and organs through multiple ways including enhanced infiltration, augmented proliferation, or decreased apoptosis [1, 19, 20]. tPA has been shown to modulate macrophage accumulation in various organs [12, 15, 21–23], such as the ischemic brain [23] and both acute [22] and chronic kidney injury [12, 15], where concomitant induction of tPA was well documented [8, 9, 24, 25]. These findings support the notion that tPA is a common endogenous factor that modulates macrophage functions and inflammatory response in multiple organ systems.

It's known that tPA in the circulation is produced by endothelial cells to maintain the homeostasis of coagulation and fibrinolysis [15, 26]. However, our recent work discovered that bone marrow-derived myeloid cells are the primary source of the interstitial induction of tPA in the obstruction-injured kidneys [15], and the endogenously induced tPA acts in an autocrine manner to promote macrophages accumulation [14, 15] and induce NF-κB-dependent renal inflammation [12]. Macrophages are heterogeneous cells that are classified into M1 and M2 subsets based on their gene expression profiles in response to different chemokines. M1 macrophages, stimulated by LPS and IFN-γ, promote inflammation and exaggerate damage, while M2 macrophages, stimulated by IL-4 or IL-13, help to resolve inflammation and promote tissue remodeling [1, 2]. In present study, we found that tPA KO mice expressed significantly higher level of M2 macrophage markers, such as Relm-α and Ym1 (Figure 1A-1D), but had dramatically decreased M1chemokine expression including IL-1β, iNOS, and IP-10 (Figure 1E-1G) than WT mice. These results are consistent with our previous finding that WT mice had dramatically higher number of TNF-α^+^CD11b^+^ or CD11b^+^F4/80^lo^CD206^−^ M1 macrophages than their KO littermates [14], and further confirm that tPA preferably promotes M1 macrophage accumulation in the fibrotic kidneys.

Several factors contribute to the renal accumulation of M1 macrophages, such as the increased number of newly activated M1 macrophages (M0 to M1), extended life span and proliferation of the existing M1 macrophages, etc. Converting from M2 macrophages also contributes to the accumulation of M1 macrophages, which may be particular important during the progression of CKD since recent observations indicate that macrophage polarization is dynamic and macrophages switch between M1 and M2 phenotypes in the damaged kidneys at different disease stages [4, 5]. The results of present study, which showed that tPA not only reduces IL-4-induced M2 makers of arginase 1 and Ym1, but also reverses the M2 chemokine profile of IL-4-treated macrophages to M1 phenotype, as demonstrated by reduced IL-10 expression and up-regulation of iNOS, TNF-α, and IL-1β (Figure 2), clearly support our hypothesis that tPA promotes macrophage M2 to M1 phenotypic change leading to the accumulation of M1 macrophages in the diseased kidneys.

We further discovered that tPA-induced macrophage M2 to M1 phenotypic change requires its receptor annexin A2 (Figure 4) but not LRP-1 (Figure 3). Intriguingly, while tPA promotes fibroblast activation, proliferation, and survival exclusively through its LRP-1 receptor [6–10, 13, 26], tPA also activates NF-κB in macrophages through annexin A2-mediated CD11b pathway [12]. Annexin A2, a member of the Ca^2+^- and phospholipid-binding protein family, is a membrane-associate protein which can only dock onto the cell membrane in the peripheral manner [27, 28]. tPA has been shown to bind to the hexapeptide LCKLSL (residues 7–12) in the N terminus of annexin A2 [29], and promote the aggregation and interaction of annexin A2 and CD11b integrin, leading to the clustering and activation of CD11b signaling in macrophages [12]. Conformational change and activation of CD11b integrin will cause the activation of its immediate downstream effector ILK, which interacts with the cytoplasmic domain of integrins through its C terminus and mediates their signaling by activating downstream mediators to phosphorylate IκB leading to its degradation and releasing of p65 and its nuclear translocation and subsequently canonical activation of NF-κB pathway [12]. Then, NF-κB triggers the transcription of proinflammatory genes and induces the M2 to M1 macrophage phenotypic change (Figure 5).

**Figure 6:**
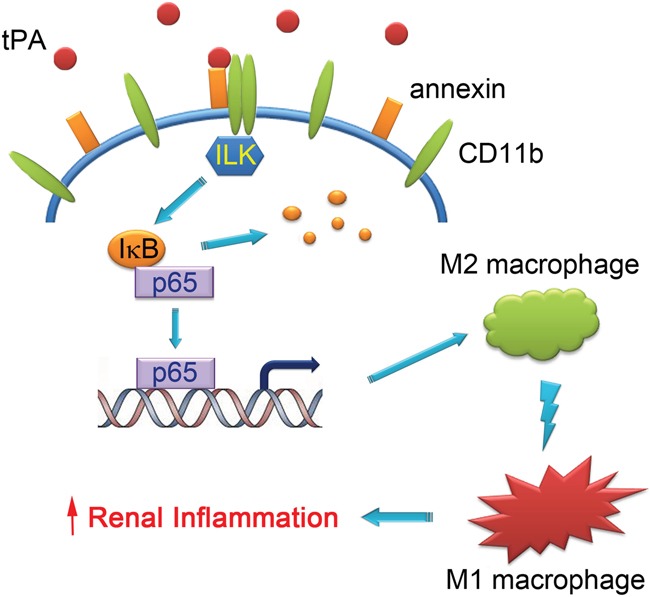
Schematic illustration of the signaling mechanism of tPA-induced macrophage M2 to M1 phenotypic change tPA binds to annexin A2 and induces the aggregation and interaction of annexin A2 and CD11b, which in turn activates ILK, phosphorylates IκB, and promotes NF-κB-mediated macrophage M2 to M1 phenotypic change, leading to increased renal inflammation.

In summary, the present work has defined the previously unrecognized function and signaling mechanism of tPA in modulating macrophage M2 to M1 phenotypic change. As shown in Figure 6, our present work, based on our previous finding that annexin A2 acts as co-receptor of CD 11b to activate ILK-mediated signaling [12], demonstrates that tPA modulates macrophage M2 to M1 phenotypic change through annexin A2-mediated ILK activation, which in turn phosphorylates IκB and activates NF-κB, contributing to the accumulation of M1 macrophages and resultant increased renal inflammation.

## MATERIALS AND METHODS

### Antibodies and reagents

Arginase 1 and GAPDH antibodies were obtained from Santa Cruz Biotechnology (Santa Cruz, CA). Relm-α antibody was bought from Novus Biologicals (Littleton, CO). Monoclonal anti-annexin A2 antibody was provided by BD Bioscience (San Diego, CA). Anti-Ym1 antibody was from Stemcell Technologies (Vancouver, BC, Canada). Mouse monoclonal anti-LRP-1 (11H4) antibody was prepared as previously described [9, 10]. The secondary HRP-conjugated antibodies, fetal bovine serum (FBS), and supplements were obtained from Fisher Scientific (Pittsburgh, PA). The non-enzymatic tPA was supplied by Molecular Innovations Inc. (Southfield, MI). Mouse recombinant IL-4 was purchased from R & D Systems (Minneapolis, MN). NF-κB specific cell permeable inhibitor peptide SN50 and its inactive control peptide SN50-con were obtained from Millipore (Billerica, MA). The Dulbecco's modified Eagle's medium (DMEM) was obtained from American Type Culture Collection (ATCC, Manassas, VA). All other chemicals of analytic grade were obtained from Sigma or Fisher Scientific unless otherwise indicated.

### Cell culture

Mouse macrophages J774.A1 were purchased from ATCC and maintained as previously described [12]. Primary bone marrow-derived macrophages were prepared and maintained as previously reported [15]. Briefly, bone marrow was extracted from the mouse femurs, followed by centrifuge at 300 rpm for 3 min. Supernatants were collected and subjected to 1000 rpm centrifuge for 10 min. Then, cell pellets were suspended and cells were seeded into non-tissue culture-treated dishes in RPMI containing 20% FBS, 10 ng/ml M-CSF, 2mM glutamine, and antibiotics. Non-adherent cells were removed by changing media every other day. After 7d, macrophages adhered to the bottom (purity > 99%) were harvested and seeded into 6-well plates. After overnight serum-free starvation, the macrophages were treated with vehicle or non-enzymatic tPA for various period of time as indicated and then collected for different assays.

### Animal model

Homozygous tPA knockout (KO) and wild-type (WT) mice on C57BL/6 background were purchased from the Jackson Laboratory (Bar Harbor, Maine) and maintained as previously described [6, 8, 10, 12]. Macrophage-specific LRP-1 KO mice (LysMCre^+^LRP^flox/flox^) and annexin A2 KO mice were generated as previously described [30–34]. Animal protocol was approved by the Institutional Animal Care and Use Committee at the Penn State University College of Medicine. Unilateral ureteral obstruction (UUO) was performed in 20-22 g gender-matched mice (3-5 mice per group) using established procedures [6, 8, 10, 12].

### siRNA transfection

Mouse LRP-1 siRNA was purchased from Thermo Scientific with target sequence as GACCAGUGUUCUCUGAAUA, whereas siRNA targeting mouse annexin A2 was obtained from Invitrogen [12]. Transient transfection of siRNAs was performed as previously described [6, 8–10, 12, 15].

### Western blot analysis

Samples were prepared and separated on 10% to 15% SDS polyacrylamide gels as previously described [6, 8–10, 12], followed by protein transfer to a PVDF membrane and incubation with various primary antibodies and HRP-conjugated secondary antibodies. Signals were visualized by a Chemiluminescent Substrate kit (Thermo Fisher Scientific).

### Quantitative RT-PCR

Total RNA was extracted and reverse transcribed into cDNA and amplified using SYBR Green PCR kit (Qiagen, Valencia, CA) as previously described [8, 12]. The sequence of the primers was reported elsewhere [35, 36]: iNOS, *Forward (F)*: CCCTGCTTTGTGCGAAGTGT, *Reverse (R)*: ATGCGG CCTCCTTTGAGC; IP-10, *F*: GGTCTGAGTGGGA CTCAAGG, *R*: CGTGGCAATGATCTCAACAC; IL-10, *F*: GCTCTTACTGACTGGCATGAG, *R*: CGC AGCTCTAGGAGCATGTG; TNF-α, *F*: CTGTAG CCCACGTCGTAGC, *R*: TTGAGATCCATGCCGTTG; IL-1β, *F*: CCCAACTGGTACATCAGCAC, *R*: TCT GCTCATTCACGAAAAGG. Relative level of mRNAs was quantified as fold increase ratio to 18s.

### Statistical analysis

All the experimental data were presented as means ± SEM. Statistical analysis of the data was performed using SigmaStat software (Jandel Scientific Software). Comparison between multiple groups was performed by using one-way ANOVA followed by the Student-Newman-Keuls test or Student *t* test between two groups. A *P* value of less than 0.05 was considered statistically significant.

## Abbreviations

tPA: tissue plasminogen activator
TNF-α: tumor necrosis factor-alpha
NF-κB: nuclear factor-kappa B
LRP-1: LDL receptor-related protein 1
IκB: inhibitory kappa B
UUO: unilateral ureteral obstruction
IL-1β: interleukin-1beta
IL-4: interkin-4
IL-10: interleukin-10
IP-10: interferon-inducible protein-10
iNOS: inducible nitric oxide synthase.
